# Outlining Core Pathways of Amyloid Toxicity in Bacteria with the RepA-WH1 Prionoid

**DOI:** 10.3389/fmicb.2017.00539

**Published:** 2017-04-04

**Authors:** Laura Molina-García, María Moreno-del Álamo, Pedro Botias, Zaira Martín-Moldes, María Fernández, Alicia Sánchez-Gorostiaga, Aída Alonso-del Valle, Juan Nogales, Jesús García-Cantalejo, Rafael Giraldo

**Affiliations:** ^1^Department of Cellular and Molecular Biology, Centro de Investigaciones Biológicas, Consejo Superior de Investigaciones CientíficasMadrid, Spain; ^2^Genomics Unit, Complutense UniversityMadrid, Spain; ^3^Department of Environmental Biology, Centro de Investigaciones Biológicas, Consejo Superior de Investigaciones CientíficasMadrid, Spain; ^4^Proteomics Facility, Centro de Investigaciones Biológicas, Consejo Superior de Investigaciones CientíficasMadrid, Spain; ^5^Department of Microbial Biotechnology, National Centre for Biotechnology, Consejo Superior de Investigaciones CientíficasMadrid, Spain

**Keywords:** amyloid proteinopathy, model amyloid disease, prionoid, systems analysis, *Escherichia coli*, membrane targeting, ROS toxicity

## Abstract

The synthetic bacterial prionoid RepA-WH1 causes a vertically transmissible amyloid proteinopathy in *Escherichia coli* that inhibits growth and eventually kills the cells. Recent *in vitro* studies show that RepA-WH1 builds pores through model lipid membranes, suggesting a possible mechanism for bacterial cell death. By comparing acutely (A31V) and mildly (ΔN37) cytotoxic mutant variants of the protein, we report here that RepA-WH1(A31V) expression decreases the intracellular osmotic pressure and compromise bacterial viability under either aerobic or anaerobic conditions. Both are effects expected from threatening membrane integrity and are in agreement with findings on the impairment by RepA-WH1(A31V) of the proton motive force (PMF)-dependent transport of ions (Fe^3+^) and ATP synthesis. Systems approaches reveal that, in aerobiosis, the PMF-independent respiratory dehydrogenase NdhII is induced in response to the reduction in intracellular levels of iron. While NdhII is known to generate H_2_O_2_ as a by-product of the autoxidation of its FAD cofactor, key proteins in the defense against oxidative stress (OxyR, KatE), together with other stress-resistance factors, are sequestered by co-aggregation with the RepA-WH1(A31V) amyloid. Our findings suggest a route for RepA-WH1 toxicity in bacteria: a primary hit of damage to the membrane, compromising bionergetics, triggers a stroke of oxidative stress, which is exacerbated due to the aggregation-dependent inactivation of enzymes and transcription factors that enable the cellular response to such injury. The proteinopathy caused by the prion-like protein RepA-WH1 in bacteria recapitulates some of the core hallmarks of human amyloid diseases.

## Introduction

Amyloids are stable and relatively simple, albeit polymorphic, structures in which peptide stretches from a given protein assemble as fibrillar β-sheet polymers of indefinite length (Riek and Eisenberg, 2016). The aggregation of proteins as amyloids is at the basis of many neurodegenerative and systemic human diseases (Eisenberg and Jucker, 2012). There are many proposed routes for amyloid cytotoxicity, including the targeting of cell membranes (Butterfield and Lashuel, 2010), co-aggregation of essential cell factors (Olzscha et al., 2011; Hosp et al., 2015), interference with intracellular traffic (Woerner et al., 2016) or overloading the protein quality triage machinery, including chaperones, the proteosome and autophagy (Hipp et al., 2014). Interestingly, mitochondria, the power engines of eukaryotic cells, have recently attracted much attention due to their involvement in several amyloid proteinopathies (Lin and Beal, 2006; Liu et al., 2015). A pioneering systems biology work reported that the disease caused in mice by distinct strains of the prion protein PrP was affecting, besides other neural and glial processes, the energetic metabolism at mitochondria (Hwang et al., 2009). Later proteomic studies revealed a major presence of mitochondrial factors co-aggregated with designed β-amyloid proteins (Olzscha et al., 2011). Targeting of mitochondria in amyloidoses has been described for α-synuclein in Parkinson’s disease (Haelterman et al., 2014), Aβ(1-40/42) and Tau in Alzheimer’s disease (García-Escudero et al., 2013), SOD1 in amyotrophic lateral sclerosis (Taylor et al., 2016), and huntingtin in Huntington’s disease (Costa and Scorrano, 2012). A ‘mitochondrial side’ in amyloid proteinopathies has thus emerged. Overall, in the mitochondria of cells undergoing amyloidosis it is clear that malfunction of the electron transport chain, with subsequent generation of reactive oxygen species (ROS), and the impairment of proton-motive force (PMF), leading to a reduction in the efficiency of ATP synthesis, are major determinants of neurodegeneration (Lin and Beal, 2006; Liu et al., 2015). Since mitochondria have bacterial endosymbiotic ancestry (Gray, 2012), it makes sense to explore if these routes for amyloid toxicity can be reconstructed and untangled in bacteria.

While much information on amyloid diseases is being derived from model systems such as mice, flies, worms, and yeast, which share genetic similarities with humans (Narayan et al., 2014), bacterial cells have not been exploited so much because, when expressed in bacteria, proteins involved in human amyloidoses aggregate as inclusion bodies (IBs) that are barely detrimental to cell fitness (Lindner et al., 2008; Winkler et al., 2010). On the other hand, bacteria use amyloids as functional tools in an extracellular context, e.g., to scaffold biofilms, as in the case of CsgA/*curli* in *Escherichia coli* (Chapman et al., 2002) or TasA in *Bacillus subtilis* (Romero et al., 2010); or to coat aerial hyphae, as chaplins/rodlins in *Streptomyces coelicolor* (Capstick et al., 2011). In particular, the complex secretion pathway for CsgA (Van Gerven et al., 2015) has been exploited as a screening platform to survey the amyloidogenic potential of proteins and to search for inhibitors of amyloidosis (Sivanathan and Hochschild, 2012). Recently, a transcriptional terminator from *Clostridium botulinum* (CbRho), has been characterized as an intracellular prion-like protein (Pallarés et al., 2016; Yuan and Hochschild, 2017). CbRho is the determinant of an epigenetically transmissible phenotype, structurally and functionally analogous to yeast prions (Liebman and Chernoff, 2012), but not a suitable model system for amyloid diseases.

Along the last 10 years, we have developed a synthetic prionoid, i.e., a cytotoxic but non-infectious prion-like protein (Aguzzi, 2009), by engineering the N-terminal ‘winged-helix’ domain (WH1) in RepA, the DNA replication protein of a bacterial plasmid (reviewed in Giraldo et al., 2016). As in the full length RepA when activated to initiate DNA replication (Giraldo et al., 2003), RepA-WH1 undergoes a conformational change *in vitro*, coupled to dissociation of protein dimers into monomers, either on transient binding to plasmid-derived DNA sequences (Giraldo, 2007; Gasset-Rosa et al., 2008) or upon templating by RepA-WH1 aggregates themselves (Fernández-Tresguerres et al., 2010). Such process enables the monomers of the highly amyloidogenic mutant A31V of RepA-WH1 to assemble into fibers composed of intertwined tubular helical protein filaments (Giraldo, 2007; Torreira et al., 2015). RepA-WH1 fibers are of amyloid nature, as indicated by Congo red binding (Giraldo, 2007), and by a net increase in the protein β-sheet contents, according to both circular dichroism (Giraldo, 2007; Torreira et al., 2015) and surface-enhanced Raman (Fernández et al., 2016a) spectroscopies. In our efforts to engineer a synthetic bacterial amyloid proteinopathy, we found that the amyloidogenicity of WH1(A31V) in *E. coli* cells can be boosted displacing its conformational equilibrium toward partial unfolding by fusing a protein to its C-terminus, distinct to the natural WH2 domain in RepA (Giraldo et al., 2003): the monomeric fluorescent protein mCherry (Fernández-Tresguerres et al., 2010; Gasset-Rosa et al., 2014; Molina-García and Giraldo, 2014). In the resulting fusion protein, for simplification hereafter WH1(A31V)-mCh (biophysically characterized in Fernández et al., 2016b), the mCherry tag has not a direct contribution to aggregation, because a fusion of mCherry to wild-type RepA-WH1 remained soluble and non-toxic in the cytoplasm (Fernández-Tresguerres et al., 2010; Molina-García and Giraldo, 2014). WH1(A31V)-mCh aggregates are vertically inheritable (from mother to daughter cells) cytotoxic particles (Fernández-Tresguerres et al., 2010), phenotypically distinct to IBs in terms of morphology, intracellular distribution and numbers, higher affinity for an amyloid-specific fluorophore, poor co-localization with IbpA (an IBs-tracer protein), and their acute cytotoxicity (Gasset-Rosa et al., 2014). WH1(A31V)-mCh propagates as at least two amyloid strains (or variants) with distinct morphologies and degrees of cytotoxicity whose interconversion is modulated by the Hsp70 chaperone DnaK (Gasset-Rosa et al., 2014), resembling the phase transitions observed in proteins involved in human amyloidoses (Giraldo et al., 2016). In coherence with the ability of DNA to promote RepA-WH1 amyloidosis *in vitro*, in *E. coli* cells amyloid precursors assemble at the bacterial nucleoid (Moreno-del Álamo et al., 2015). Interestingly, a recent study reveals that the full length RepA protein, through its WH1 domain, assembles as a functional amyloid at the bacterial nucleoid to physically couple plasmid DNA replication origins, thus preventing premature re-initiation events (Molina-García et al., 2016). Binding of WH1(A31V)-mCh to the bacterial cell membrane *in vitro*, or to lipid vesicles having an acidic phospholipid composition, has revealed that lipids also promote the amyloidogenesis of the protein and its assembly as transmembrane pores *in vitro* (Fernández et al., 2016b), as many proteins involved in human amyloidoses do (Butterfield and Lashuel, 2010).

Here we have explored the pathways for the amyloid cytotoxicity triggered by the RepA-WH1 prionoid in *E. coli*, aiming to outline a simplified chain of events shedding light on the molecular mechanism(s) operating in human amyloidoses, which so far have revealed as extremely complex and refractory to untangle. In bacteria undergoing WH1(A31V)-mCh amyloidosis, membrane targeting is operational as the primary mechanism of damage to cells both under aerobic and anaerobic conditions. Combined transcriptomic and interactomic studies reveal that up to 501 genes or proteins are potentially involved in amyloidosis, forming part of over 40 functional clusters of which a significant fraction contributes to the following major cellular processes: carbon metabolism, NADH and (Fe-S)-dependent oxido-reduction, transport through the inner membrane, iron uptake, (Fe-S) clusters assembly, nucleic acids metabolism, cell division and responses to stress, in particular detoxification of ROS. Several of these targets were then functionally validated. The primary loss in PMF leads to a substantial depletion of the ATP pool and, due to the consequent reduction in the intracellular levels of iron, enhances the expression of NdhII. This dehydrogenase generates H_2_O_2_ by auto-oxidation, while several of the proteins involved in detoxifying peroxide reduce their expression or co-aggregate with the prionoid, thus sensitizing bacteria toward oxidative stress, which ultimately stalls cell division and leads to cell death. RepA-WH1 amyloidosis provides a unique window to survey the essential landscape of a general amyloid proteinopathy, endorsing this prion-like protein as a generic, minimal bacterial model of amyloid disease.

## Materials and Methods

### Bacterial Strains and Culture Conditions

Expression of either WH1(A31V)-mCh or WH1(ΔN37)-mCh was performed from low copy-number plasmids under the control of the P*tac* promoter (described in Gasset-Rosa et al., 2014). A construct just carrying the mCherry protein (Molina-García and Giraldo, 2014) was used as a control. As bacterial host, the reduced genome *E. coli* K-12 strain MDS42 *recA* (Pósfai et al., 2006) was used in all experiments because it provides a simplified ‘chassis’ carrying the essential metabolic and regulatory pathways. Bacterial cells were transformed with the plasmids and grown at 37°C in 200 mL of rich LB medium (supplied with 2 mg⋅mL^-1^ thymine and 100 μg⋅mL^-1^ ampicillin) with good aeration in 1 L Erlenmeyer flasks. Induction was achieved by adding IPTG to 0.5 mM when cultures reached OD_600__nm_ = 0.2. Cells were harvested at various post-induction intervals, washed and, for the transcriptomic and interactomic analyses, immediately frozen in liquid nitrogen and then transferred to -70°C for storage. Cells (4⋅10^8^-3⋅10^9^, depending on the assay) were collected from at least three independent culture replicas.

### Microscopy

Bacterial cells were observed with a Nikon Eclipse 90i microscope, equipped with a CFI PLAN APO VC 100x (NA 1.40) oil immersion objective and a Hamamatsu ORCA-R^2^ CCD camera. For mCherry fluorescence, a 543/22 nm excitation and 593/40 nm emission filter and 200 ms exposures were used. Differential interference contrast (DIC) shots (100 ms) were also captured. Images were analyzed using the NIS-Elements AR software (Nikon). Bacterial culture aliquots were fixed in formaldehyde and mounted on poly-L-lysine coated slides, as described in Fernández-Tresguerres et al. (2010).

### Luciferase Assays Monitoring Intracellular ATP Levels

In a first approach, *E. coli* bulk cultures, expressing or not the RepA-WH1 prionoid, were grown as indicated above. Upon IPTG induction, every 30 min 4⋅10^8^ bacterial cells were harvested and lysed. The levels of ATP were determined *in vitro* using the ATP Bioluminiscence assay HSII (Roche), which is based on the requirement of ATP by firefly luciferase to process luciferin and emit light at 562 nm. Samples were dispensed in 96 wells black-walled microtiter plates and read-outs acquired in a TD-20/20 Turner Designs luminometer. Plots were corrected to the dry weight of cells.

In a second approach, bioluminiscence was monitored in real time in microscale cultures. In this assay, bacteria carried the vector for the expression of WH1(A31V)-mCh (Gasset-Rosa et al., 2014) plus mini-CTX-*lux* (Becher and Schweizer, 2000), a plasmid constitutively expressing the *Photorhabdus luminescens luxCDABE* operon from the kanamycin promoter. Cultures in LB (no antibiotics added) at OD_600__nm_ = 0.05 were fractioned in 200 μL aliquots and displayed in 96 well, flat bottom and black-walled, Grenier Chimney plates. When required, IPTG was supplied to 0.5 mM at the beginning of the experiment and each plate was then incubated in a Tecan infinite M200 PRO plate reader for 24 h at 37°C. At 30 min intervals, plate was agitated for 5 s (2 mm amplitude) and the following variables were sequentially measured: absorption (at 600 nm, 9 nm bandwidth), luminiscence (1 s integration time) and fluorescence (546 nm excitation, 9 nm bandwidth; 600 nm emission, 20 nm bandwidth; 25 flashes for 20 μs). Data were normalized to the OD_600__nm_ values. For each experiment, three replicas were set up.

### Determination of the Intracellular Concentration of Iron

Bacterial cultures were grown as specified above and iron concentration in the cell pellets was determined based in the ability of ferrozine to form a complex with Fe^2+^ that absorbs light at 562 nm (Honn et al., 2012). Volumes proportional to the cell densities in the cultures (1.0 OD_600__nm_≈ 8⋅10^8^ bacteria) were taken at time intervals and then cells were harvested, washed and resuspended in PBS buffer. Bacteria were lysed with 100 μL NaOH and then neutralized with 100 μL of 10 mM HCl. Cell lysates were incubated with 100 μL of protein uncoupling solution (0.7 M HCl, 2.25% KMnO_4_) for 2 h at 60°C. Then samples were incubated for 30 min with 100 μL of 6.5 mM ferrozine, 6.5 mM neocuproine, 2.5 M ammonium acetate, 1 M ascorbic acid, and the mixture was centrifuged for 30 s at 13,000 rpm. A_562__nm_ was measured for all supernatants in a Varioskan Flash (Thermo scientific) plate reader. The values of absorption obtained were normalized to the dry cell weight. The whole set of samples was processed at the same time for each replica of the assay to achieve reproducibility.

### Viability of Bacteria Expressing the Prionoid under Aerobic vs. Anaerobic Conditions

Cells were grown aerobically, as described above, or anaerobically in LB medium supplemented with 10 mM nitrate as terminal electron acceptor and 5 mM cysteine as reducing agent. Bottles with 20 ml of LB medium, as well as the nitrate and cysteine stock solutions (100x), were flushed with N_2_, sealed with rubber stoppers and aluminum foil and then autoclaved. Then bottles were introduced in an anaerobic chamber (Forma anaerobic system 1029 S/N, Thermo Scientific) in which the air was continuously interchanged with a mixture of N_2_ and biogas (10% H_2_, 5% CO_2_ and 85% N_2_). The nitrate and cysteine supplements and the bacterial inocula were injected into the bottles through the stopper and cultures were incubated at 37°C under low shaking conditions (150 rpm). Bacterial growth was monitored as OD_600__nm_. Serial dilutions of the cultures at initial-log phase were plated on LB-agar, which had been supplemented with nitrate and cysteine and left to stand at the anaerobic chamber for at least 24 h before usage. The rest of bacteria were induced with 0.5 mM IPTG and further grown until reaching mid-log and then early stationary phase, when serial dilutions were also plated. Incubations were carried out at 37°C under aerobic or anaerobic conditions and then colony forming units (cfu) per mL were counted. These experiments were performed in triplicate.

### Transcriptomic Analysis of the Response of *E. coli* to the RepA-WH1 Prionoid

WH1(A31V/ΔN37)-mCh expression was induced under aerobiosis as indicated above. For RNA purification, the RNeasy kit (Qiagen) was used, followed by in-column DNaseI digestion (RNase-free, Roche; 10 μL, 2 h at 37°C). The purity of the RNA preparation was assessed first through AGE (0.8% agarose in TAE buffer, samples pre-incubated in 50% formamide buffer, at 95°C for 2 min) and then in a Bioanalyzer 2100 RNA chip (Agilent). Final RNA concentrations ranged between 0.5 and 0.75 μg⋅mL^-1^ and their absorption ratios at 260/280 nm were between 2.13 and 2.45. Equal amounts of each RNA sample were retro-transcribed to DNA using random sequence oligonucleotide hexamers as primers. Template RNAs were then degraded with NaOH and cDNAs were labeled using TdT DNA polymerase and ddUTP-biotin. Labeled cDNAs were hybridized on GeneChip^®^ *E. coli* Genome 2.0 arrays (Affymetrix), which span 10,000 probesets from the pangenome of four *E. coli* strains (including MG1655, the parental for MDS42) and casted on a Fluidics Station 450 (Affymetrix) at 45°C for 16 h. Arrays were washed, stained with phycoerythrin-conjugated streptavidin and then fluorescence emission at 570 nm was digitized in a GeneChip^®^ Scanner 3000 7G (Affymetrix), as specified by the supplier. Microarrays were identically processed for three independent biological replicas. Data were normalized with the RMA algorithm (Affymetrix Expression Console software) and analyzed using the Babelomics software package (Medina et al., 2010). Statistical analysis of the results was performed through the limma *t*-test with Benjamini–Hochberg’s FDR correction: genes with false discovery rates (FDR) ≤ 0.05 were classified as significantly induced/repressed. Data were manually filtered to discard low score (background) genes not present in the MDS42 genome (Pósfai et al., 2006). Genes with A31V/ΔN37 expression ratios either higher than 2 or lower than 0.5 were selected as the fraction of the *E. coli* genome preferentially expressed or repressed, respectively, in response to WH1(A31V)-mCh amyloidosis. Microarray data are available at the Gene Expression Omnibus database (GEO) under the accession number GSE69517.

### Interactomic Analysis of the Co-aggregation of *E. coli* Proteome with RepA-WH1

After induction of MDS42 cells carrying either WH1(A31V)-mCh or WH1(ΔN37)-mCh (see above), 13 A_600__nm_ units were processed at 0.5, 1, and 2.5 h by lysing the cell pellets with 1.5 mL of 20 mM Hepes⋅NaOH pH 6.0, 0.1 M NaCl, 0.5% sulfobetaine 12 (SB-12), 0.5% Na-deoxycholate, 1 mM EDTA, 50 μg.mL^-1^ RNaseA, plus a protease inhibitors pill (Roche). Cell lysates were then centrifuged at 12,000 rpm for 1 h at 4°C. Pellets were resuspended in 1.5 mL of the same buffer, but with 1.0 M NaCl and no RNaseA, and they were sonicated (Branson ultrasonic homogenizer, thin tip) for 30 s and centrifuged as above. The sedimented fraction was resuspended in 250 μL of 20 mM Hepes⋅NaOH pH 6.0, 0.1 M NaCl, 1 mM EDTA and this suspension was then carefully layered on a discontinuous sucrose (20–40% in the same buffer) cushion and centrifuged overnight at 12,000 rpm and 4°C. Pellets were subsequently resuspended in Laemmli buffer (x2), their component proteins analyzed by SDS-PAGE (10% polyacrylamide) and then gels stained with Coomassie blue. Proteins bands over and below WH1(A31V/ΔN37)-mCh were excised, cut into pieces and digested in gel (50 mM NH_4_HCO_3_, overnight at 30°C) with bovine trypsin (12.5 μg⋅mL^-1^). Peptides were extracted in acetonitrile and 0.5% trifluoroacetic acid, cleaned through a ZipTip (C18 matrix; Millipore) and resuspended in 0.1% formic acid, 2% acetonitrile (buffer-A). Peptides were processed as described (Barderas et al., 2013). Briefly, peptides were trapped in a C18-A1 ASY-Column (Thermo Scientific) and, upon elution, loaded into a Biosphere C18 column (NanoSeparations). A 125-min gradient (250 nL⋅min^-1^) from 0 to 35% buffer-B (0.1% formic acid in 100% acetonitrile), followed by steps to 45% (15 min) and 95% (10 min), was developed in a NanoEasy HPLC coupled to a nanoelectrospray ion source (Proxeon). Mass spectra (*m*/*z* 300–1700) were generated in an LTQ-Orbitrap Velos MS (Thermo Scientific) in the positive ion mode and acquired with a target value of 1,000,000 at a resolution of 30,000 (*m*/*z* 400). The 15 most intense ions were selected for collision-induced fragmentation in the linear ion trap with a target value of 10,000 and normalized collision energy of 38%. Raw MS files were searched with the SEQUEST algorithm (Eng et al., 1994) against the *E. coli* MDS42 proteome (UniProt). Peptides were validated with Percolator (Spivak et al., 2009), scoring as positive those proteins with ≥3 identified peptides per target, or with a peptide spectrum match (PSM) value ≥ number of identified peptides and XCorr > 3. Proteins represented in the mass spectra by a single peptide were not considered, except when PSM > 3. If present in both datasets, proteins classified as co-aggregated with ΔN37 were then subtracted from those listed for A31V. The whole procedure was repeated for three independent biological replicas. Proteins found at least twice as preferentially co-aggregated with the A31V variant were selected as the fraction of the *E. coli* proteome co-aggregated with WH1(A31V)-mCh.

### Comparison of the Transcriptomic and Interactomic Datasets

The lists of genes preferentially induced/repressed or co-aggregated with WH1(A31V)-mCh, but not with the ΔN37 variant, were processed in parallel in a similar way, including Boolean algebra analysis with Venny^1^, classifying genes (or proteins) as *early* when present just in the 0.5 h dataset or when found both at 0.5 and 1.0 h, middle when exclusively placed in the 1.0 h dataset, and late when present at 2.5 h alone or both at 1.0 and 2.5 h. Gene ontology (GO) functional classification was performed with the EcoCyc database (Keseler et al., 2013). The curated transcriptomic and interactomic datasets were finally crossed using the STRING 10.0 tool (Szklarczyk et al., 2015) to get a comprehensive set of the functional pathways and protein clusters involved in WH1(A31V)-mCh amyloidosis.

### HPLC Analysis of Metabolic Succinate and Acetate

Bacterial cultures were grown as indicated above. One mL aliquots were collected at post-induction intervals, cells removed by centrifugation at 13,000 rpm for 5 min, and the culture supernatants were processed through 0.2 μm filters and stored at -80°C. Samples were analyzed in triplicate, as described in Felpeto-Santero et al. (2015). Twenty microliter samples were injected into an Aminex HPX-87H column (Bio-Rad) coupled to a Gilson HPLC system. Elution was performed at 0.6 mL⋅min^-1^ in 5 mM H_2_SO_4_. Identification and quantitation of the acetate and succinate peaks were carried out using 32 Karat (v. 8.0; Beckman-Coulter). Metabolite concentrations were extrapolated from the elution profiles of calibrated solutions of acetate and succinate. Plots were corrected according to the dry weight of bacterial pellets.

### Assay for Inhibition by ROS of Bacterial Growth on Agar

Bacterial cultures were grown to OD_600__nm_ = 0.4 and 400 μL plated on LB agar with 100 μg⋅mL^-1^ ampicillin and 0.5 mM IPTG. When indicated, plates were supplemented with ascorbic acid to 1.5 mM to neutralize hydrogen peroxide. Sterile filter paper disks (Whatman, 0.5 mm ∅) were embedded with 0.001% H_2_O_2_ or 0.0025% (w/v) paraquat (Sigma), and then laid on the plates and cultured at 37°C overnight. For the *Δndh* SLC22 cells (Woodmansee and Imlay, 2002) H_2_O_2_ was tested up to 0.5%. Areas of the inhibition halos were estimated on photographs, subtracting the area of the paper disks.

## Results

### WH1(A31V)-mCh Targets the Inner Cell Membrane, Hampering PMF-Dependent Transport and ATP Synthesis

**FIGURE 1:**
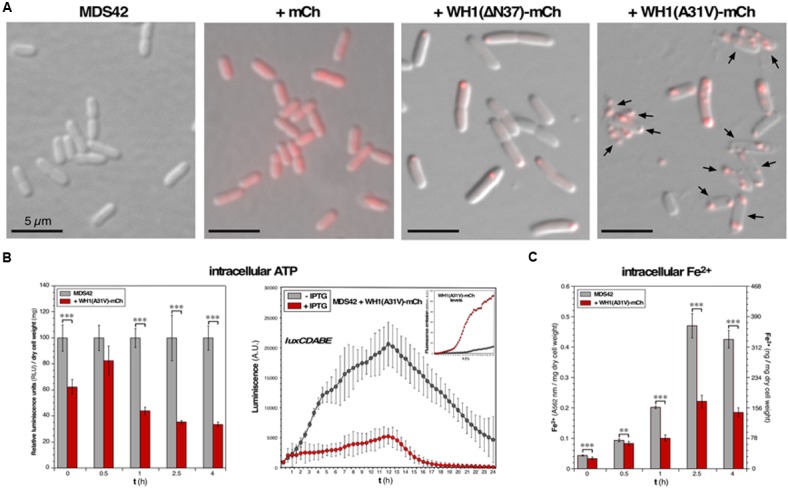
**Functional assessment of membrane integrity in bacteria undergoing the WH1(A31V)-mCh amyloidosis. (A)** Images overlaying red epifluorescence and DIC micrographs of *E. coli* MDS42 cells (*left*), or the same strain expressing (2.5 h post-induction) mCherry (*center*), or WH1(A31V)-mCh (*right*). Arrows: bacterial cells having lost turgor. **(B)** *Left*: Estimation of the ATP levels in whole cell bacterial lysates. Mean values (bars) and SDs (whiskers) from 3 biological replicas are displayed, normalized to the values measured for prionoid-freed cells. *Right*: Effect of the expression of WH1(A31V)-mCh on the luminiscence of live *E. coli* cells that constitutively expressed bacterial luciferase. The levels of the prionoid were monitored in parallel by measuring the red fluorescence emission (inset). Dots: mean values from three independent culture wells; whiskers: SDs. **(C)** Intracellular iron levels in naïve bacteria or upon expression of WH1(A31V)-mCh. Iron uptake is impaired in *E. coli* cells undergoing amyloidosis. *Y* axes represent the read-out of the ferrozine assay (light absorption at 562 nm; *left*) and its conversion to the amount of ferrous iron (*right*), both expressed per mg of dry cell mass. Data from 6 biological replicas. One-way ANOVA statistical significance analysis, followed by Tukey’s pairwise difference test, was performed for panels B and C. ^∗∗^*p* < 0.01; ^∗∗∗^*p* < 0.001.

The hyper-amyloidogenic A31V variant of RepA-WH1 (Giraldo, 2007) becomes metastable and highly cytotoxic upon fusion to the monomeric red fluorescent protein mCherry (Gasset-Rosa et al., 2014). The resulting prion-like protein, WH1(A31V)-mCh, has the ability to assemble pores in model lipid vesicles that mimic the *E. coli* inner membrane, thus leaking their contents while not suffering lysis (Fernández et al., 2016b). Expression of WH1(A31V)-mCh in the *E. coli* K-12 MDS42 strain resulted, when bacteria were observed at the microscope (**Figure 1A**), in a significant proportion of ‘ghost’ cells. In a clear indication for a weakened integrity of the membrane, cells lost their normal turgor but, as for the vesicles, did not lyse retaining their large size components such as the nucleoid and the prionoid aggregates. On the contrary, bacteria expressing the soluble mCherry reporter did not show any difference in morphology compared with the parental strain.

The integrity of the cell membrane is critical to the generation of a PMF, which drives ATP synthesis by the membrane-bound ATP synthase. If, as observed *in vitro* (Fernández et al., 2016b), WH1(A31V)-mCh targets the inner membrane through pore formation, membrane integrity is expected to be compromised, with the subsequent reduction of PMF-dependent processes such as ATP synthesis. To test this hypothesis, we measured the concentration of ATP in cell lysates from bulk *E. coli* cultures grown aerobically in rich medium, by measuring the *in vitro* activity of the ATP-dependent firefly luciferase: a progressive reduction in luminiscence (up to ≈ 70% at ≥ 2.5 h) was observed upon the expression of the prionoid (**Figure 1B**, left). In a different bioluminiscence assay, based on the constitutive *in vivo* expression of the bacterial *luxCDABE* operon, ATP was consumed by LuxD in the synthesis of the substrate for the LuxAB luciferase. In this case, the expression of WH1(A31V)-mCh also led to a net reduction (by ≈ 75%) in luminiscence emission (**Figure 1B**, right). Both results point to a significant drop in the intracellular amount of ATP and thus are consistent with a scenario of compromised bioenergetics.

We then focused on iron uptake to probe the integrity of the inner cell membrane further. Iron is an essential co-factor in many reactions central to aerobic metabolism, especially those involving the oxidoreduction of substrates. Being a scarce resource, Gram-negative bacteria have evolved siderophores, scavenger molecules with high-affinity and specificity for iron (Frawley and Fang, 2014). Once synthesized, siderophores are secreted through both membranes and, after extracellular coordination of the Fe^3+^ ion, they are internalized in a process that is dependent on both PMF and ATP consumption. Upon reduction to Fe^2+^, the metal is released in the cytoplasm to be assembled as mononuclear iron or as (Fe-S) clusters in metalloproteins. So, the intracellular level of iron provides another estimation of the ability of the cell membrane to support transport and thus on its integrity. We determined the intracellular concentration of iron across the time course of the induction of WH1(A31V)-mCh (**Figure 1C**). Ferrous iron increased steadily for 2.5 h in both the naïve and prionoid-expressing cells but was significantly lower (about 50% after 1 h) in the cells undergoing amyloidogenesis.

The findings reported in this section are consistent with a reduction in PMF, and thus in ATP synthesis, due to prionoid-elicited leakage through the inner membrane.

### Viability of *E. coli* Is Reduced by WH1(A31V)-mCh under Both Aerobic and Anaerobic Growth

**FIGURE 2:**
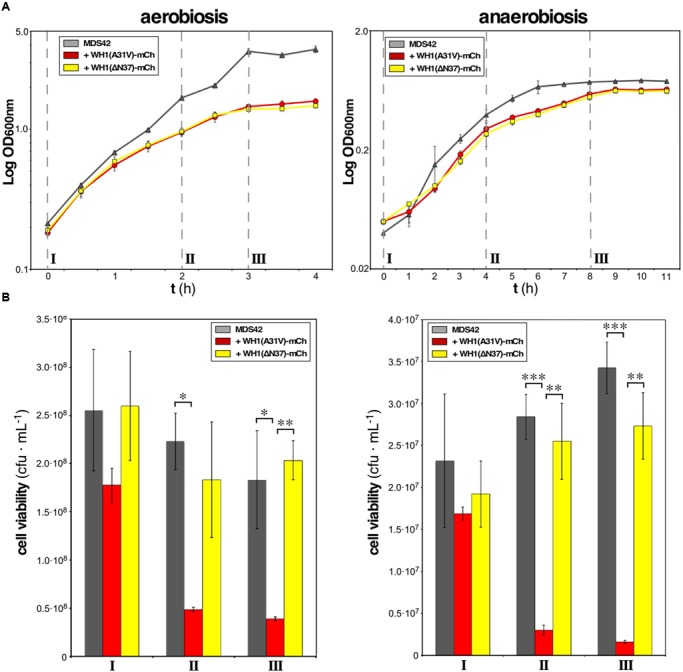
**WH1(A31V)-mCh expression is cytotoxic under both aerobiosis and anaerobiosis. (A)** Semi-logarithmic growth curves, in aerobic (*left*) or anaerobic (*right*) conditions, of naïve *E. coli* cells expressing or not either of the two variants (A31V or ΔN37) of WH1-mCh. Cultures were grown in triplicate. **(B)** Viability of bacteria from cultures in **(A)** was checked by plating on LB-agar equal cell numbers at three stages: pre-induction (I), mid-exponential (II), and early stationary (III) phases. Colony forming units (cfu) per mL were counted after incubation under aerobiosis or anaerobiosis. Bars: mean values; whiskers: SDs. One-way ANOVA statistical significance analysis, followed by Tukey’s pairwise difference test, was performed (^∗^*p* < 0.05; ^∗∗^*p* < 0.01; ^∗∗∗^*p* < 0.001).

Targeting of the inner bacterial membrane by amyloids is a mechanism of cytotoxicity that must be operating whatever is the final acceptor in the electron transport chain. *E. coli* is a facultative anaerobe. Therefore, it made sense to survey whether prionoid cytotoxicity occurred under aerobic and/or anaerobic growth conditions. This study was carried out in parallel upon the expression of two distinct mutant variants of RepA-WH1, A31V and ΔN37, both fused to mCherry: while the former is hyper-amyloidogenic and highly cytotoxic (Giraldo, 2007; Gasset-Rosa et al., 2014), the latter, lacking the amyloidogenic stretch in RepA-WH1, aggregates as conventional IBs and has a milder cytotoxicity (Gasset-Rosa et al., 2014). Relative to the maximum optical density reached by the cells freed of the prionoid, under aerobic conditions a 60% reduction was observed for the cultures expressing the prionoid (**Figure 2A**, left), whereas in anaerobic growth such reduction was just 20% (**Figure 2A**, right). The viability of cells was then checked at three points of the respective growth curves: pre-induction, middle exponential and early stationary phases. As expected from the cell densities achieved (**Figure 2A**), the number of colonies per mL of culture, once plated on agar, was an order of magnitude higher for bacteria grown under aerobiosis than for those in anaerobiosis (**Figure 2B**). The most noticeable difference was that, under both physiological conditions, the expression of WH1(A31V)-mCh drastically reduced (to 10–20%) the viability of the bacterial population, whereas WH1(ΔN37)-mCh did not in a significant way. These results indicated that the expression of WH1(A31V)-mCh indeed is cytotoxic. However, the ΔN37 mutant has no deleterious effect and thus the reduction in growth observed for this variant (**Figure 2A**) must be a burden on fitness imposed by the formation of IBs. As *E. coli* is usually grown under aerobic conditions, and these actually are closer to the environment for human cells undergoing amyloidoses, the rest of the experiments reported here were carried out in aerobiosis.

### Global Transcriptional Response of *E. coli* to the Expression of the WH1(A31V)-mCh Prionoid

Transcriptomic analysis provided clues on how bacteria react to the expression of the prionoid downstream of its primary target, the inner cell membrane. In a subtractive gene expression approach using microarrays, WH1(ΔN37)-mCh IBs were used as a reference set for WH1(A31V)-mCh, thus suppressing from the output list genes involved in the unspecific cellular response to protein aggregation/IBs, such as molecular chaperones and quality control proteases (Winkler et al., 2010). This focused the study on features specific for the acute cytotoxicity of the prionoid. The same *E. coli* strain used above, MDS42 (Pósfai et al., 2006) was selected again as host bacteria because its reduced genome, devoid of non-essential genes, simplified the transcriptomic analysis. In previous studies (Fernández-Tresguerres et al., 2010; Gasset-Rosa et al., 2014), time-lapsed fluorescence microscopy allowed us to characterize 30 min as the post-induction time interval in which WH1(A31V)-mCh aggregates started to become evident in a substantial fraction of the cells, and 2.5 h as the point where cytotoxicity was notorious in the form of stalled cell division, increased filamentation and subsequent cell death, which became dominant at *≥*4 h. We therefore carried out the analysis at 0.5 and 2.5 h, plus an intermediate sampling point (1 h).

**FIGURE 3:**
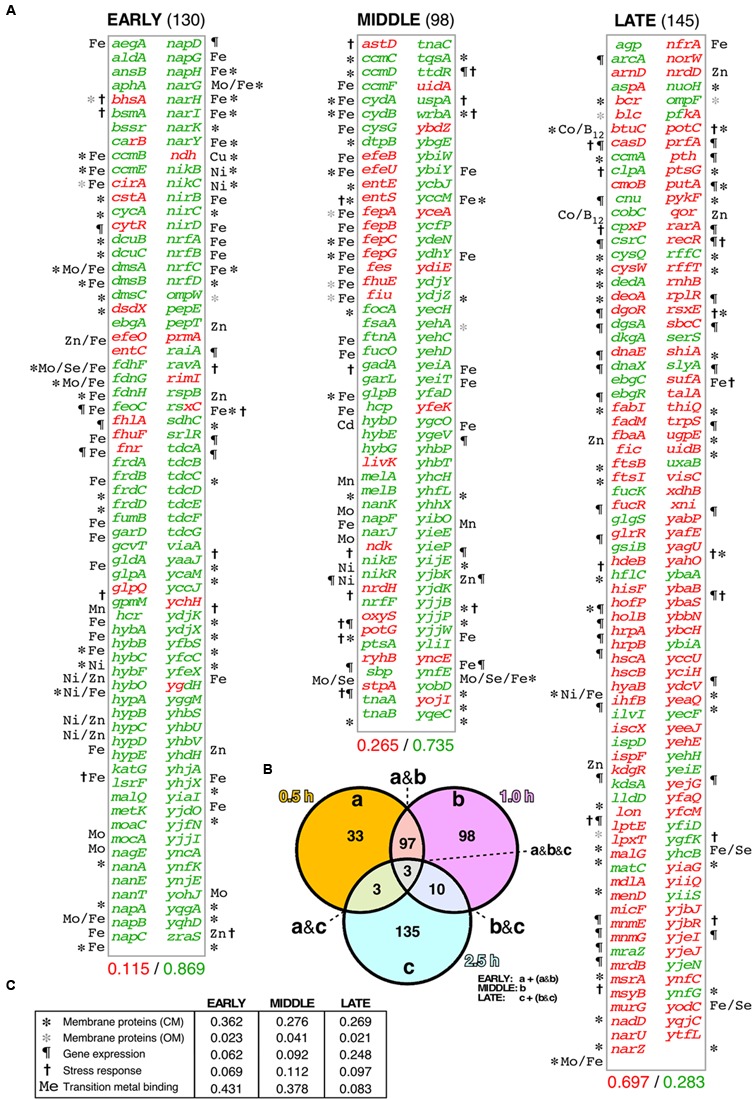
**Differential transcriptomic response of *E. coli* cells to the A31V or the ΔN37 mutants of RepA-WH1. (A)** List of the genes found to be at least two-fold induced (red) or repressed (green) in cells bearing WH1(A31V)-mCh at the *early* (*left*), middle and late (*right*) time intervals, as defined in **(B)**, compared with bacteria carrying the ΔN37 variant (**Supplementary Dataset S1**). Symbols correspond to the GO terms, as described in **(C)**. The decimal fraction of over-expressed *vs*. over-repressed genes is printed below. **(B)** Venn diagram showing the temporal distribution and number of genes whose expression levels were consistently found altered upon induction of WH1(A31V)-mCh. **(C)** Temporal distribution in five main functional gene ontology (GO) terms of the genes preferentially expressed/repressed with WH1(A31V)-mCh.

Cells from bacterial cultures expressing either WH1(A31V)-mCh or WH1(ΔN37)-mCh were harvested at the three indicated post-induction times. Total RNA samples were hybridized with DNA microarrays that probed the complete transcriptome of *E. coli*. Differentially expressed genes from the comparison of the A31V and ΔN37 datasets were classified as induced (≥2-fold expression level in A31V *vs.* ΔN37, i.e., A31V/ΔN37 ratio ≥ 2.0; in red in **Figure 3A**) or repressed (≥2-fold expression level in ΔN37 *vs.* A31V, i.e., A31V/ΔN37 ratio ≤ 0.5; in green in **Figure 3A**) (**Supplementary Dataset S1**). Genes were then grouped (**Figure 3B**) as *early* expressed (130 genes), if the levels of their mRNAs were altered only at 0.5 h, or both at 0.5 and 1.0 h; *middle* (98 genes), if they appeared in the list exclusively at 1 h; and *late* (145 genes), if they were altered after both 1.0 and 2.5 h, or just at 2.5 h. These three classes comprised most of the genes, with just a few being excluded due to their ubiquitous presence or to their simultaneous clustering at the initial and final datasets. Overall, the *E. coli* transcriptome indicated an initially repressive response to the expression of WH1(A31V)-mCh (86.9% genes differentially repressed *vs*. 11.5% induced, compared to ΔN37, in the *early* group class), with a progressive reactivation of the gene expression program (73.5% genes repressed *vs*. 26.5% induced; *middle*), which finally became dominant (28.3% genes repressed *vs*. 69.7% induced; *late*).

Functional annotation of the genes differentially affected by WH1(A31V)-mCh expression revealed (**Figure 3C**) a major fraction encoding membrane-located proteins, closely followed by metalloproteins, especially at shorter times. Other functional groups included stress response genes and DNA/RNA-binding proteins, such as transcriptional regulators, which became significant in the *late* class accounting for the observed reactivation of gene expression. *Early* repressed genes included many dehydrogenases, terminal reductases and enzymes of the anaerobic metabolism having in common iron as cofactor. This was also the case for the catalase *katG*, a major detoxifier of H_2_O_2_ (Imlay, 2008, 2013) and the most repressed gene in the whole transcriptomic dataset (**Supplementary Dataset S1**). Among the few differentially overexpressed *early* genes, were notable those for the synthesis and transport of siderophores (iron uptake pathway), such as *cirA*, *efeO*, *entC*, *fhlA* and *fhuF* (Frawley and Fang, 2014). This response was in agreement with the observed reduction in the levels of intracellular iron (**Figure 1C**). The inductions of the H_2_O_2_-responsive gene *ychH* (Lee et al., 2009) and *ndh* were also significant. The latter encodes NdhII, the major NADH-dehydrogenase in exponentially growing *E. coli* cells (Messner and Imlay, 1999), which is typically induced in response to limiting concentrations of intracellular iron (Folsom et al., 2014). On the contrary, other dehydrogenases effective in generating a PMF (Unden and Bongaerts, 1997) were repressed. The highest *early* expression was achieved for *fnr*, which encodes the oxygen-labile Fe-dependent transcription factor regulating the switch between aerobic and anaerobic metabolism (Myers et al., 2013). The *middle* group class also showed the increased expression of iron uptake genes (*efeBU*, *entES*, *fepABCG*, *fes*, *fhuE*, *fiu*) and of RyhB, an antisense RNA that is the main repressor of genes encoding iron-metalloenzymes (Massé et al., 2005), whereas the gene encoding ferritin (*ftnA*), a major Fe-storage protein, was repressed. In addition, the expression of genes responsible for the response against oxidative stress was enhanced through the regulatory antisense RNA OxyS. Expression of genes for (deoxy)ribonucleotide triphosphate synthesis, such as *ndk* and *nrdH*, and importers of anti-oxidant polyamines like *potG*, was enhanced at the transition to the *late* group class, when cell viability was already severely compromised. Other functional *late* processes included the assembly of (Fe-S) clusters (*hscAB*, *iscX*, *sufA*; being the latter the second highest expressed gene), the responses to osmotic (*putA*) and acidic (*hdeB*) stresses, and elements of the genome maintenance (*deoA*, *holB*, *recR*, *rarA*) and cell division (*ftsBI*, *mrdB*, *murG*) machineries. Relevant to the latter response, it has been recently found that filamented *E. coli* cells with compromised membrane integrity overexpress cell division genes (Sánchez-Gorostiaga et al., 2016).

### Assessment of the Fraction of the *E. coli* Proteome Co-aggregated with the WH1(A31V)-mCh Prionoid

**FIGURE 4:**
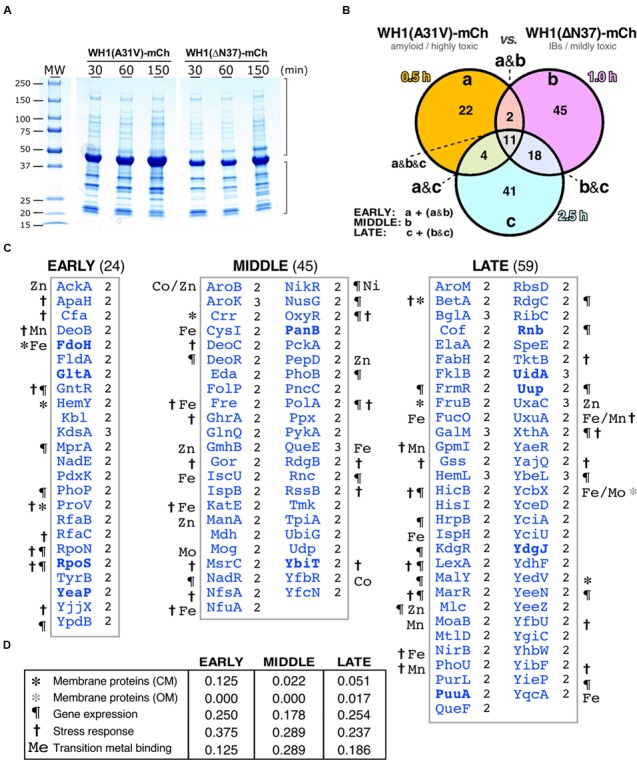
**Differential interactomics in *E. coli* cells expressing the A31V or the ΔN37 mutants of RepA-WH1. (A)** SDS-PAGE showing the aggregated protein fraction from bacteria expressing either WH1(A31V)-mCh or the WH1(ΔN37)-mCh mutant. **(B)** Venn diagram displaying the temporal distribution of the proteins found exclusively co-aggregated with the A31V variant of the prionoid. **(C)** Lists of the proteins found co-aggregated with WH1(A31V)-mCh, but not with ΔN37, in at least two of the three biological replicas (right-hand notation: 2/3; **Supplementary Dataset S2**) at the indicated time slots **(B)**. Symbols correspond to the GO terms, as described in panel D (to compare with transcriptomics, see **Figure 3C**). The eight proteins in common with the transcriptomic dataset (**Figure 3**) are in boldface. **(D)** Temporal distribution across five main functional gene ontology (GO) terms of the proteins preferentially co-aggregated with WH1(A31V)-mCh.

The loss of function caused by the assembly of proteins into amyloids is usually associated with co-aggregation of a subset of the cell proteome leading, if not to cytotoxicity itself, to the aggravation of the proteinopathic condition (Olzscha et al., 2011; Hosp et al., 2015). To explore whether the amyloidogenesis of RepA-WH1 led to the differential co-aggregation of particular proteins from the *E. coli* proteome, we undertook the purification and characterization (**Figure 4**) of the aggregated protein subset from bacteria expressing either WH1(A31V)-mCh or its milder version WH1(ΔN37)-mCh, at the same time intervals previously surveyed through genomic approaches (**Figure 3**). Protein aggregates from three independent cultures were first purified by centrifugation through discontinuous gradients of sucrose, and subsequent separation of the sediment by means of SDS-PAGE (**Figure 4A**). Gel tracks were split into slices and then proteins were digested *in situ* with trypsin. The resulting peptides were extracted and analyzed by nano-scale HPLC combined with mass spectrometry (ESI-MS). Peptides were identified in sequence databases and then classified (**Figure 4B**) following the same criteria used for the microarray studies (**Figure 3B**).

Proteins identified as preferentially co-aggregating with the WH1(A31V)-mCh prionoid, but not with the WH1(ΔN37)-mCh IBs, (**Figure 4C** and **Supplementary Dataset S2**) were less than those inferred from the transcriptomic studies (**Figure 3**): 24 proteins were consistently found (i.e., they were present with a significant score in at least two out of three biological replicas) at the *early* time interval of expression, 45 at the *middle* class and 59 at the *late* group (**Figure 4C**). Overall, functional annotation revealed that membrane proteins were underrepresented in the datasets, as expected for cytoplasmic aggregates, whereas proteins involved in the response to different types of stress were overrepresented, albeit decreasing along the time course, with the gene expression and transition metal binding functional classes ranking second and third, respectively (**Figure 4D**). The master regulator of the general stress response RpoS (σ^38^/σ^S^) (Battesti et al., 2011) was among the factors aggregating at the *early* time interval. In the *middle* group, the RpoS inhibitor RssB was found together with a number of proteins involved in the response to oxidative stress such as its master regulator OxyR (Aslund et al., 1999; Seo et al., 2015), the alternative catalase KatE, and the glutathione reductase Gor (Imlay, 2008, 2013). The (Fe-S) cluster scaffolding proteins IscU and NfuA (Jang and Imlay, 2010) were also placed in this subset. In the *late* class, BetA, an enzyme for the synthesis of the osmo-protectant betaine (Lamark et al., 1996) and DNA repair enzymes such as RdgC and XthA were identified. It is noteworthy that several enzymes in the glycolytic (pyruvate kinase II, PykA; triosephosphate isomerase, TpiA), TCA (malate dehydrogenase, Mdh) and mixed acid fermentation (acetate kinase, AckA) pathways appeared aggregated with RepA-WH1(A31V)-mCherry at the *early* and *middle* subsets.

### Combining Transcriptomics and Interactomics Highlights Central Pathways in WH1(A31V)-mCh Amyloidosis

The lists of genes up/down regulated in the transcriptomic analysis (**Figure 3**) and of proteins found as preferentially co-aggregated with WH1(A31V)-mCh (**Figure 4**) were then compared. The assumption was that differential gene expression and protein sequestration might be independent contributors to RepA-WH1 amyloidosis and thus complementary, rather than overlapping, views to the core cellular processes involved in the ‘disease.’ Indeed only eight proteins, and their respective genes, were present in both ‘omic’ datasets (1.6% of a total of 501).

**FIGURE 5:**
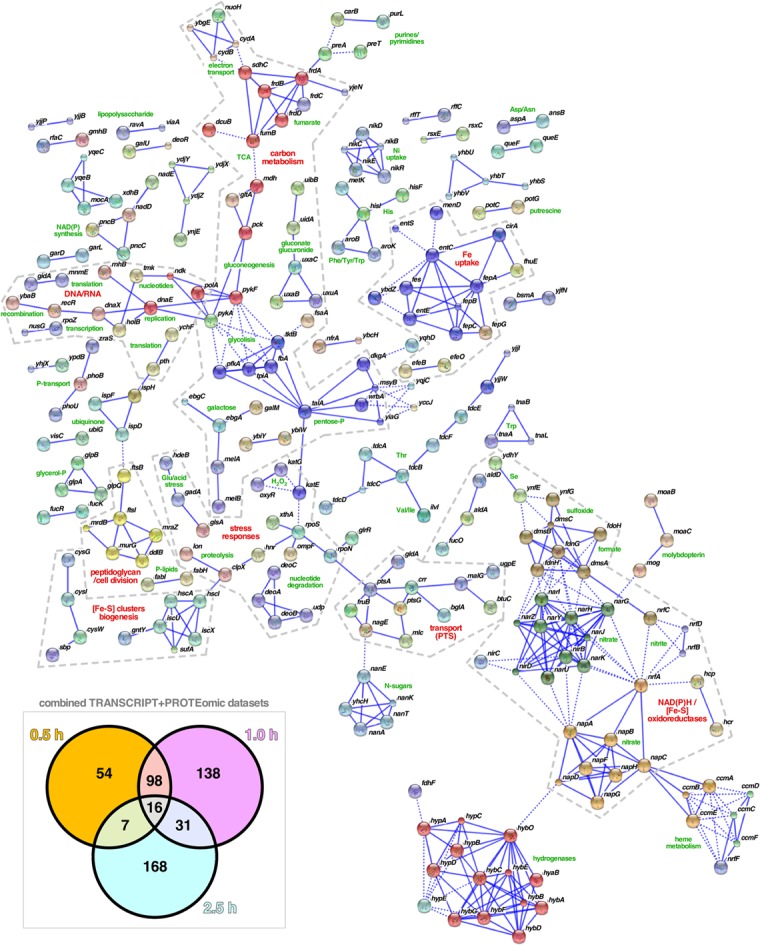
**Functional clustering of the genes/proteins related to RepA-WH1 amyloidosis.** The combined dataset of 512 genes and proteins involved in the response of *E. coli* to the amyloidosis caused by the WH1(A31V)-mCh prionoid (*box*: Venn plot; see **Figures 3**, **4**) were functionally classified using STRING 10.0 (Szklarczyk et al., 2015). All available prediction methods were active, and a stringent 0.925 confidence interval and a K-means clustering procedure were applied. The number of interactors displayed was limited to 5 and nodes that appeared disconnected were removed from the plot. The inferred function for each cluster is printed (green), while higher order functional groups (red) are highlighted within dashed boundaries.

**FIGURE 6:**
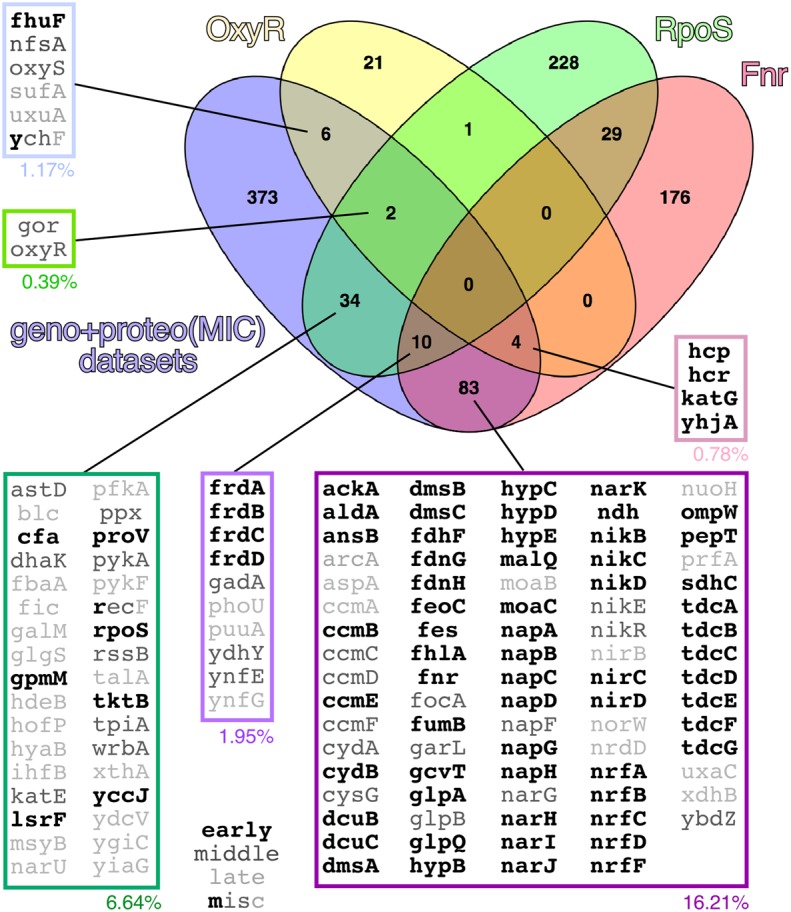
**Venn diagram showing the set of genes having OxyR, RpoS and/or Fnr as direct regulators in the combined transcriptomic and interactomic datasets.** In the expanded boxes, genes/proteins are grouped according to a Boolean analysis, with indications of the percentage they represent of the whole experimental dataset and, in gray scale characters, their occurrence along the experimental time course (early, middle, late or multiple). Other analyzed regulators (not shown) included ArcA (6.25% of the genes/proteins in the combined datasets), Fur (5.27%), RpoN (3.91%), RpoE (3.13%), PhoB (1.37%), LexA (0.78%) and CpxR (0.59%). Regulatory networks were defined according to the EcoCyc database (Keseler et al., 2013).

Network analysis of the combined set of genes or proteins allowed their assignment to over 40 functional clusters (**Figure 5**), which could be broadly grouped into eight core functions: hydrocarbon metabolism, respiration [i.e., electron transport, NAD(P)H oxidoreductases and hydrogenases]; nucleotide/phosphate and nucleic acids metabolism; transport through membranes; cell division; iron uptake; (Fe-S) clusters biogenesis; and response to various stresses (with a focus on detoxification of hydrogen peroxide). In terms of the regulatory response(s) to the aggregation of WH1(A31V)-mCh, the analysis of the combined transcriptomic and interactomic datasets revealed that the master regulators of the transcriptional switches in response to oxygen levels, Fnr (Myers et al., 2013), and to general stress, RpoS (Battesti et al., 2011), were directly controlling the expression of substantial fractions (16.21 and 6.64%, respectively, with 1.95% regulated by both) of the genes linked to RepA-WH1 amyloidosis (**Figure 6**). Other transcription factors, such as OxyR, ArcA, Fur, RpoN/E, PhoB, LexA or CpxR, fell well behind.

The assays presented above converge in a picture of damage to the bacterial inner membrane by WH1(A31V)-mCh with the subsequent reduction in PMF-dependent transport of metabolites and co-factors, such as iron. Limiting iron levels would promote the expression of the NdhII dehydrogenase that, under aerobic conditions, would generate ROS, while a battery of the proteins responsive to oxidative stress would become disabled by co-aggregation with the prionoid. With the aim of validating this sketch of the bacterial amyloidosis, we undertook additional functional assays in *E. coli* cultures expressing WH1(A31V)-mCh or WH1(ΔN37)-mCh under the same conditions surveyed through the genomic and interactomic approaches.

### WH1(A31V)-mCh Amyloidosis Leads to Impaired Carbon Metabolism

Replenishment of ATP from ADP has other sources apart from ATP synthase: the reactions of the central carbon metabolism and substrate-level phosphorylation. Upon the impairment of ATP synthase activity due to the disruption of PMF by membrane leakage, cells would become dependent on less efficient metabolic fluxes (see above; **Figure 1B**). Thus, glycolysis must be enhanced, as suggested by the observed induction of the pyruvate kinase gene (*pykF*) in cells expressing WH1(A31V)-mCh (**Figure 3A**). However, this does not seem to be the case probably due to the co-aggregation with the prionoid of triosephosphate isomerase (TpiA; **Figure 4C**). Therefore, other alternative sources for ATP regeneration were explored.

**FIGURE 7:**
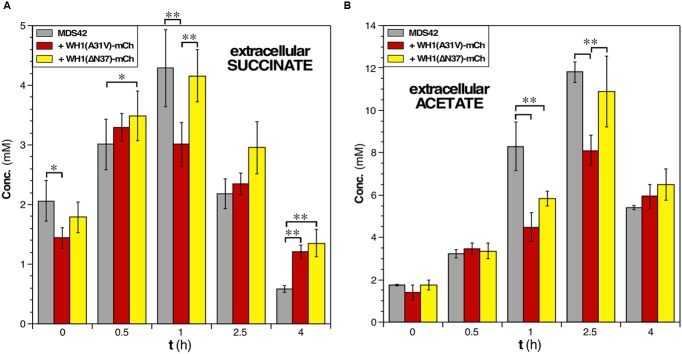
**Assessment of the concentrations of key metabolites in *E. coli* cells undergoing the RepA-WH1 amyloidosis. (A)** Determination of the extracellular concentration of the TCA cycle intermediate succinate in cultures of naïve cells or expressing the two variants of WH1-mCh. Histogram plot the concentrations (areas under HPLC peaks) of the metabolite at the indicated post-induction times, upon correction for the dry cell mass of the bacteria in the cultures. The mean values from 3 biological replicas and the SDs are shown. **(B)** The extracellular concentration of the fermentative end metabolite acetate, determined as indicated in A. One-way ANOVA plus Tukey’s pairwise difference test were used to check for statistical significance. ^∗^*p* < 0.05; ^∗∗^*p* < 0.01.

Determination by HPLC of the extracellular levels of succinate, a key intermediate in the TCA cycle, showed that expression of the prionoid resulted in a net 30% decrease in this metabolite after 1 h (**Figure 7A**). This probably reflects an early blockage in the TCA cycle due to the co-aggregation of malate dehydrogenase (Mdh) with WH1(A31V)-mCh (**Figure 4C**), besides the impossibility to regenerate NAD^+^ at the level of the electron transport chain. Interestingly, succinate levels remained more elevated for the ΔN37 than for the A31V variant of RepA-WH1, resembling the behavior of wild-type cells.

In rapidly growing *E. coli* cells, potentially leading to oxygen-limiting conditions, extra reducing power and ATP are usually generated through mixed-acid fermentation, whose products (acetate in particular) are secreted to the medium but, upon reaching stationary phase, are imported to be further metabolized (Förster and Gescher, 2014). Such double-way metabolic flux was observed in the HPLC determination of the levels of acetate in the culture medium of bacteria not expressing the prionoid (**Figure 7B**). However, upon the expression of WH1(A31V)-mCh a significant decrease (up to 30%) in the levels of acetate was detected at 2.0–2.5 h post-induction, suggesting a reduction in the production of ATP at substrate-level phosphorylation. This fact could be due to the co-aggregation of acetate kinase (AckA) in the *early* interactomic dataset (**Figure 4C**), but also to an impaired flux through glycolysis and fermentation (see above). On the contrary, the acetate profile for cells expressing WH1(ΔN37)-mCh was closer to that found in control cells. We attempted to measure the levels of other metabolites, but the results were inconclusive due to the high variability between replicas.

Overall, these results are consistent with a primary disruption in PMF by the RepA-WH1 prionoid, reinforced by a net reduction in the fluxes through both central carbon metabolism and mixed acid fermentation.

### WH1(A31V)-mCh Amyloidosis Sensitizes Bacterial Cells to Hydrogen Peroxide

Our transcriptomic analysis on *E. coli* cells in aerobiosis had shown that *katG*, the gene coding the major catalase/peroxidase at the exponential growth phase (Imlay, 2008, 2013), was the most repressed at the *early* time interval upon WH1(A31V)-mCh expression (**Figure 3A** and **Supplementary Dataset S1**). In addition, interactomics had identified the alternative stationary phase catalase KatE as significantly trapped in the intracellular aggregates of the prionoid (**Figure 4C** and **Supplementary Dataset S2**) (Imlay, 2008, 2013). These observations meant that *E. coli* cells suffering from WH1(A31V)-mCh amyloidosis must exhibit increased sensitivity toward stress by hydrogen peroxide. On the contrary, no superoxide dismutase (SodABC) showed altered expression, or differential co-aggregation, upon expression of the A31V or ΔN37 variants. Therefore, bacterial cells undergoing the WH1(A31V)-mCh amyloidosis must not be differentially sensitive to superoxide.

**FIGURE 8:**
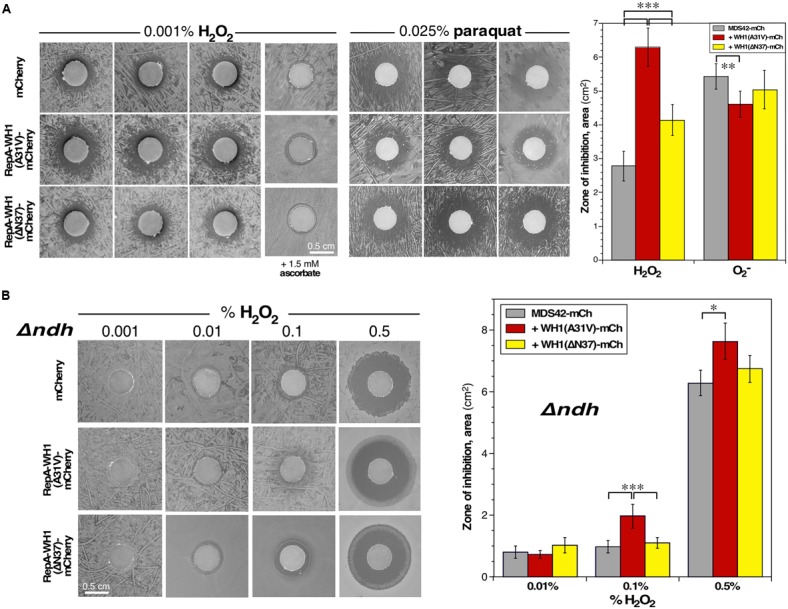
**Bacteria expressing the RepA-WH1 prionoid show NdhII-enhanced sensitivity to H_2_O_2_. (A)** *Left*: Zonal inhibition assay of H_2_O_2_, embedded in paper disks, on the growth of a lawn of *E. coli* expressing WH1(A31V/ΔN37)-mCh, or a control mCherry reporter. Three independent replicas are displayed. Ascorbate, a ROS scavenger, has a neutralizing effect (last column). *Middle*: A similar assay, but using instead the $O_{2}^{•-}$ generator paraquat. *Right*: Quantitation of the mean areas of inhibition. Data were extracted from 12 biological replicas (whiskers, SDs). Cells expressing WH1(A31V)-mCh exhibit a higher sensitivity to H_2_O_2_ than those expressing ΔN37 or, most notably, the control mCherry. There is no difference in sensitivity toward superoxide. **(B)** *Left*: Zonal inhibition assays of the growth of a Δ*ndh E. coli* strain, expressing either WH1(A31V/ΔN37)-mCh, or a mCherry control, including different concentrations of H_2_O_2_ in the disks **(A)**. *Right*: Mean areas of inhibition. Data were collected from 8 biological replicas (whiskers, SDs). Hyper-sensitivity to H_2_O_2_ as linked to RepA-WH1, notably to its A31V variant, seems to source from NdhII because *Δndh* cells can stand higher levels of peroxide. Statistical significance was estimated by one-way ANOVA, followed by Tukey’s pairwise difference test. ^∗^*p* < 0.05; ^∗∗^*p* < 0.01; ^∗∗∗^*p* < 0.001.

We thus challenged bacteria with diluted H_2_O_2_ (**Figure 8A**, left) or paraquat (**Figure 8A**, middle), a generator of superoxide radicals ($O_{2}^{•-}$), and tested their effects in a zonal growth inhibition assay on agar plates. Briefly, lawns of cells expressing the control marker mCherry, or its fusion to WH1(A31V) or WH1(ΔN37), were seeded just before laying filters pre-embedded with the oxidizing agents. Quantitation of the areas of the inhibition halos revealed (**Figure 8A**, right) that the expression of WH1(A31V)-mCh correlated with a net hindrance of bacterial proliferation by H_2_O_2_ (125% increase in area, compared with the mCherry control), an inhibition higher than that observed upon expression of the ΔN37 variant (43% increase). However, no significant differences were appreciated when the three bacterial strains were treated with paraquat. As expected, the inhibitory effect of H_2_O_2_ was relieved by the inclusion of a reducing agent (ascorbate) in the medium (**Figure 8A**, left).

These results support an impairment, dependent on WH1(A31V)-mCh, of the cellular response against the oxidative stress caused by H_2_O_2_.

### NdhII Likely Is a Source of ROS in *E. coli* Cells Undergoing WH1(A31V)-mCh Amyloidosis

Growth of bacteria under aerobic conditions generates vast amounts of ROS (up to μM intracellular concentrations) (Messner and Imlay, 1999). In cells undergoing WH1(A31V)-mCh amyloidosis, the only differentially induced NADH-dehydrogenase at the *early* stage was NdhII (encoded by *ndh*; **Figure 3A**). NdhII is expressed in response to limiting levels of iron (Folsom et al., 2014) and generates ROS through the auto-oxidation of its FAD cofactor (Messner and Imlay, 1999; Woodmansee and Imlay, 2002; but see Seaver and Imlay, 2004). So, an increase in oxidative stress was expected as a side effect of the observed rise in the intracellular levels of NdhII, with the possible consequence of a higher sensitization of cells to exogenous oxidizing agents. To test this hypothesis, zonal growth inhibition assays with H_2_O_2_ were performed in a *Δndh* (null) mutant *E. coli* background (Woodmansee and Imlay, 2002). The results (**Figure 8B**, left) revealed a net reduction in the sensitivity of the mutant bacteria to the additional stress imposed by exogenous hydrogen peroxide: up to a 500-fold increase in H_2_O_2_ concentration was required to get inhibition halos with an area close to that observed in the *ndh*^+^ background, while keeping the trend of the higher sensitivity of bacteria expressing WH1(A31V)-mCh (**Figure 8B**, right).

These results suggest that induction of the alternative dehydrogenase NdhII is a relevant source of ROS in bacteria undergoing WH1(A31V)-mCh amyloidosis, to the point of overtaking proteins involved in detoxifying H_2_O_2_, a defense line already feeble due to their co-aggregation with the prionoid (**Figure 4C**).

## Discussion

**FIGURE 9:**
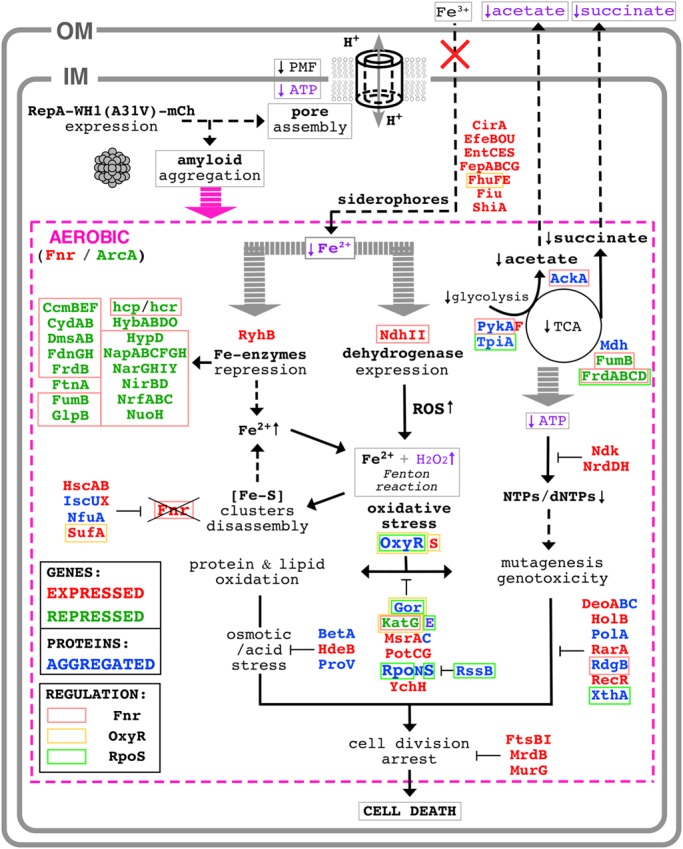
**Scheme of the molecular pathways leading to bacterial cell death by the RepA-WH1 prionoid.** The intracellular WH1(A31V)-mCh prionoid drills pores through the bacterial inner membrane, thus triggering a proteinopathy. For further details on the downstream aerobic pathways (magenta), see text. Proteins whose expression was found enhanced (red) of reduced (green) in an attempt to counteract the effects of amyloidosis are indicated, as well as those co-aggregated (blue) with the prionoid. The latter are expected to be functionally defective, worsening the course of the ‘disease.’ The functional assays performed here to validate pathways picked out by the ‘omic’ approaches are typed in purple. Three master regulators of the response to stress (Fnr, OxyR and RpoS) appear engaged in WH1(A31V)-mCh amyloidosis, and thus the proteins they regulate are displayed boxed (**Figure 6**).

**FIGURE 10:**
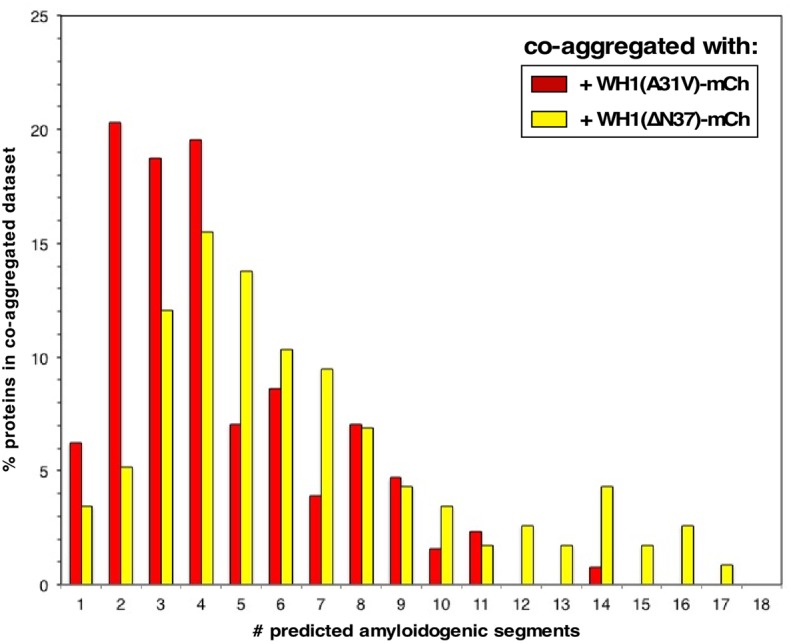
**Distribution of the number of amyloidogenic sequences in the proteins exclusively co-aggregated with WH1(A31V)-mCh (red) or with the ΔN37 mutant (yellow) (**Figure 4** and **Supplementary Dataset S2**).** The sequences of the proteins found to co-aggregate with the A31V variant (128 entries) and those found in ΔN37 aggregates (116 proteins) were analyzed with the WALTZ tool to predict amyloidogenic stretches in proteins (Maurer-Stroh et al., 2010).

Through a combination of complementary approaches, we have outlined a chain of events leading to the death of bacterial cells caused by the RepA-WH1 prionoid in its hyper-amyloidogenic mutant variant A31V (**Figure 9**). To our knowledge, this is the first attempt to globally address in bacteria the pathways for amyloid toxicity. It is noteworthy that all the effects reported here as due to WH1(A31V)-mCh stand out from those caused by the expression of WH1(ΔN37)-mCh, a deletion variant lacking the major amyloidogenic stretch in the protein (L_26_VLCAVSLI_34_; Giraldo, 2007), which is milder in terms of cytotoxicity and forms IBs distinct to the prionoid aggregates. Thus, the observed alterations in the transcriptome, the fraction aggregated in the proteome (interactomics) and in the physiology of bacteria expressing the prionoid can be accounted for as genuinely elicited by protein amyloidosis, not by unspecific protein aggregation. For the sequences of the proteins differentially co-aggregated with WH1(A31V)-mCh, the distribution of predicted aggregation-prone stretches clusters around 2–4 per protein, while those aggregated with WH1(ΔN37)-mCh show a more spread, bimodal distribution around 4–5 and 14 stretches (**Figure 10**). A similar trend had been described while comparing the sequences of proteins involved in amyloid diseases with those aggregating as IBs, and it was attributed to the ability of amyloids to assemble on the basis of a defined and discrete number of interfaces, instead of the multiple, barely specific contacts established in IBs (Conchillo-Solé et al., 2007). The amyloidogenic stretch in WH1(A31V) might capture, while assembling, other amyloidogenic segments in the proteome, whereas WH1(ΔN37) would entrap less selectively other proteins, through multiple hydrophobic interactions, while they are folding. It is remarkable that the entries in the transcriptomic and interactomic datasets show little overlap, as expected if co-aggregation with, and transcriptional response to, the prionoid were additive players in RepA-WH1 amyloidosis.

The synthetic bacterial model ‘proteinopathy’ caused in *E. coli* by WH1(A31V)-mCh would be initiated upon targeting the prionoid to the inner cell membrane, in coherence with recent results on the assembly by this protein of pores through lipidic vesicles *in vitro* (Fernández et al., 2016b). The assembly of membrane pores is common to several amyloidogenic proteins involved in human neurodegenerative diseases (Butterfield and Lashuel, 2010). Although leakage through the pores of small molecule cofactors essential for the respiratory chain cannot be excluded, membrane drilling necessarily leads to disruption of PMF. A shutdown of PMF is consistent with the observed reduction in the coupled transport of iron (**Figure 1C**), and would hinder the activity of transmembrane dehydrogenases (including Complex I: NdhI/NuoA-N). Membrane damage is the primary physical mechanism of toxicity and it is operational in both aerobiosis and anaerobiosis (**Figure 2**).

In a scenario of low PMF due to a leaky inner membrane, respiration would heavily depend on the alternative NADH dehydrogenase NdhII, which is induced (**Figure 3A**) in response to low intracellular levels of iron (Folsom et al., 2014), as found upon WH1(A31V)-mCh expression (**Figure 1C**). NdhII usually is the most active dehydrogenase under exponential aerobic growth (Messner and Imlay, 1999), but the expression of the prionoid seems to potentiate further such central role. However, NdhII has its disadvantages. Firstly, because it does not create a PMF, NdhII is poorer than NdhI in terms of generation of ATP (Unden and Bongaerts, 1997). Rather than depending on the normal end of the respiratory chain (F_1_/F_0_ ATPase), bacteria would then rely on the glycolytic pathway to recharge ATP from ADP. However, the energetic metabolism in bacteria undergoing the WH1(A31V)-mCh amyloidosis is affected by a decrease in the flux from glycolysis to the TCA cycle (**Figure 7A**), and also in substrate level phosphorylation (i.e., acetate fermentation; **Figure 7B**). Such restrictions in metabolic fluxes might be imposed by the co-aggregation with the prionoid of key enzymes such as TpiA, PykA, Mdh and AckA (**Figure 4C**). There is an attempt to regenerate the pool of nucleotide triphosphates through the overexpression of the nucleotide di-phosphate kinase Ndk, but this must be inefficient because this enzyme uses ATP. Secondly, as a by-product of NdhII activity, vast amounts of ROS (both superoxide and hydrogen peroxide) are generated (Messner and Imlay, 1999; Seaver and Imlay, 2004). NdhII seems to be a relevant source of oxidative stress in cells undergoing WH1(A31V)-mCh amyloidosis, as revealed by the enhanced sensitivity to a challenge with exogenous H_2_O_2_ in *ndh*^+^ (**Figure 8A**) over *Δndh* genetic backgrounds (**Figure 8B**). Superoxide dismutases (SodAB) seem to be unaltered in the transcriptome (**Figure 3**) and are absent from the co-aggregated proteome (**Figure 4**), thus they cope with the transmutation into H_2_O_2_ of the $O_{2}^{•-}$ radicals generated by NdhII. However, the oxidative stress-responsive catalases are either hyper-repressed (KatG) or co-aggregated with WH1(A31V)-mCh (KatE), thus converting H_2_O_2_ in a major problem. The repression of *katG* can be due to the aggregation of OxyR, which is the master transcriptional activator of the genes responsive to oxidative stress (Aslund et al., 1999; Seo et al., 2015; Imlay, 2015b). This would also explain why other members of the OxyR regulon, such as *ahpCF*, *dps* and *fur*, do not show up in differential transcriptomics (**Figure 3A**). It is noteworthy that simultaneous disabling of several detoxifying enzymes, as implied here from the sequestering of OxyR, KatE and Gor into the aggregates, has been postulated as a requirement in sensitizing bacteria against ROS (Imlay, 2015a).

Another consequence of an uncontrolled generation of ROS is the H_2_O_2_-promoted disassembly of (Fe-S) clusters. In particular, the transcriptional regulator Fnr is a sensible target in oxidative stress (Myers et al., 2013). Fnr turnover seems to be assured through an increase in its transcription, as *fnr* is in fact the most expressed *early* gene (**Supplementary Dataset S1**). In the combined genomic and interactomic datasets, up to 62 *early* genes/proteins (40.79% of 152) are directly regulated by Fnr, whereas this number goes down to 20 (14.49% of 138) and 13 (6.53% of 199) in the *middle* and *late* groups, respectively (**Figure 6**). Therefore, Fnr likely is the transcription factor responsible for triggering the transcriptomic response of *E. coli* cells to the expression of the WH1(A31V)-mCh prionoid.

In the global transcriptional response to the WH1(A31V)-mCh amyloidosis (**Figure 3** and **Supplementary Dataset S1**) it is noteworthy the induction of genes encoding siderophores, iron scavengers that are first exported and then internalized through the two *E. coli* membranes to fulfill their role (Frawley and Fang, 2014). Since such transport is actually impaired (**Figure 1C**) due to the reduction in PMF and ATP levels (**Figure 1B**) imposed by membrane leakage, siderophore expression most likely is futile. Bacteria also seem to react to iron starvation by repressing a plethora of metabolic enzymes having this metal as a cofactor, through the expression of the small antisense RNA RyhB (Massé et al., 2005). A second source that may increase the availability of iron is disassembly of the essential (Fe-S) clusters that, as mentioned above for Fnr, is enhanced by oxidative stress and would be counteracted by induction of proteins involved in chaperoning their assembly, such as IscX, HscAB and SufA (Jang and Imlay, 2010) (**Figure 3A**). However, this route might be compromised because IscU and NfuA were found co-aggregated with the prionoid (**Figure 4C**).

In the late stage of the synthetic amyloidosis caused in *E. coli* by the WH1(A31V)-mCh prionoid, the concurrence in the cytoplasm of H_2_O_2_ and some freed iron, the latter from dismantled mononuclear Fe-enzymes and (Fe-S)-containing proteins and the reduced levels of a major Fe-storage protein (ferritin, FtnA), would result in the generation of hydroxyl radicals through Fenton chemistry. These radicals lead to massive oxidation of lipids, proteins and DNA, and the outcome of genotoxicity (Al Mamun et al., 2012). Although this final sequence of events remains to be experimentally addressed, it seems that there is a last attempt of counteracting such a ‘terminal multi-systemic failure’ through expression of a battery of enzymes in the response pathways to oxidative, osmotic and acidic stresses, as well as involved in DNA repair and cell division (**Figure 3**). However, such desperate efforts had no apparent success, since bacteria were committed to death since the initial targeting of the cell membrane.

The sequence of events sketched above for the WH1(A31V)-mCh amyloidosis in *E. coli* (**Figure 9**) has some points in common with the phenotypic responses that this bacterium assembles to confront, besides oxidative stress (Myers et al., 2013; Seo et al., 2015), other kind of injuries such as acidic pH and osmotic/salt stresses (Weber et al., 2005), high pressures (Malone et al., 2006), iron starvation (Folsom et al., 2014), phage/envelope stress (Bury-Moné et al., 2009), stress-induced mutagenesis (Al Mamun et al., 2012), and antibiotic treatment (Foti et al., 2012). Probably the mechanism closest to that proposed here for the RepA-WH1 prionoid is found for cationic antimicrobial peptides, which target cell membranes as amyloids do and trigger a similar ROS response (Choi et al., 2015). It is noteworthy that some of the routes outlined here for amyloid toxicity, in particular those relative to membrane bioenergetics and central metabolism, have been described as relevant for bacteria to become ‘persisters’ against external stress, including antibiotics (Harms et al., 2016). However, as a viable state, persistence can be overcome thanks to the stress-responsive genes regulated by RpoS, while in WH1(A31V)-mCh amyloidosis this transcription factor is early sequestered through aggregation (**Figure 4C**). The cytotoxicity elicited by the bacterial prionoid thus appears to be a class of its own.

Interestingly, the scenario outlined for the bacterial WH1(A31V)-mCh amyloidosis (**Figure 9**), far from being an oddity emerging from a synthetic construction, might resemble some mitochondrial routes in a wide spectrum of human amyloid diseases (Lin and Beal, 2006). Although mammalian cells lack the alternative NdhII dehydrogenase, Aβ, Tau and α-synuclein induce the generation of ROS by Complex I (NdhI) in neurons and glial cells, with the impairment of transport through membranes and a reduction in ATP generation (Liu et al., 2015). WH1(A31V)-mCh amyloidosis also shares significant similarities with the cytotoxicity pathways described for PrP in transmissible spongiform encephalopathies: (i) the generation of ROS in glial cells by NAD(P)H oxidase (NOX2) in the respiratory chain (Sorce et al., 2014); and (ii) the expression of genes involved in iron homeostasis (Hwang et al., 2009).

The data presented here on the molecular pathways of the ‘proteinopathy’ caused in bacteria by the prionoid WH1(A31V)-mCh outline a minimal, reductionist sketch for a general amyloid disease at the cellular level that, as main core dysfunctions, would imply: (i) protein aggregates targeting the bacterial (or mitochondrial) inner membrane, linked to impaired transport and respiration; and (ii) the subsequent iron-enhanced generation of cytotoxic ROS, coupled to co-aggregation driven inactivation of key detoxifying proteins. Adding to the discoveries made along the last decade on this prion-like protein, the results reported here empower bacteria as model systems of amyloidoses, providing a versatile platform to test interventions aiming to counteract intracellular amyloid proteinopathies in more complex systems.

## Author Contributions

LM-G, MM-d, PB, ZM-M, MF, AA-d and AS-G performed the research; JG-C and JN designed the transcriptomic and metabolite analyses, respectively; all authors analyzed data; RG conceived the project, integrated the results and wrote the paper.

## Conflict of Interest Statement

The authors declare that the research was conducted in the absence of any commercial or financial relationships that could be construed as a potential conflict of interest.

## Footnotes

1 http://bioinfogp.cnb.csic.es/tools/venny/index.html

## Supplementary Material

DATASET S1 **Genes found in the transcriptomic analysis either induced or repressed in response to the expression of WH1(A31V)-mCh.** Genes were annotated (see **Figure 3**) as either induced (> +2.0 fold ratio, in red) or repressed ( < -2.0, in green) according to the ratios between the levels in bacteria expressing this hyper-amyloidogenic variant of the prionoid and those found in cells expressing WH1(ΔN37)-mCh. Each sheet displays a different point in the experimental time course and includes, for every entry, its probe ID, probability values, false discovery rates (FDR; ≤0.05) and functional annotations. Statistical significance was determined on three biological replicas.

DATASET S2 **Proteins found co-aggregated with WH1(A31V)-mCh.** Each sheet displays the proteins identified as enriched in the aggregates formed by this hyper-amyloidogenic variant of the prionoid, but not by WH1(ΔN37)-mCh, at a different point in the experimental time course and in an independent biological replica (see **Figure 4**). For every entry, its reference number in the UniProt database and a functional description are displayed. Mass spectrometry parameters such as the calculated score, the % of coverage of the protein sequence by the identified peptides and their numbers, and the peptide spectrum match (PSM) value are also shown, together with the number of amino acids residues and the theoretical molecular weight of the targets.
